# Supplementary material for: Causal interplay between lactose intolerance and gut microbiota: a combined bidirectional Mendelian randomization and in vivo validation study
Source: Front Nutr. 2026 Jun 1;13:1803337. doi: 10.3389/fnut.2026.1803337 (PMC13265576; doi:10.3389/fnut.2026.1803337)
Supplement: Supplementary file 1 [file Data_Sheet_1.zip › supplementary materials/Forward/leave one out cross validation/ebi-a-GCST90027765.finngen_R12_E4_LACTONAS.pdf]

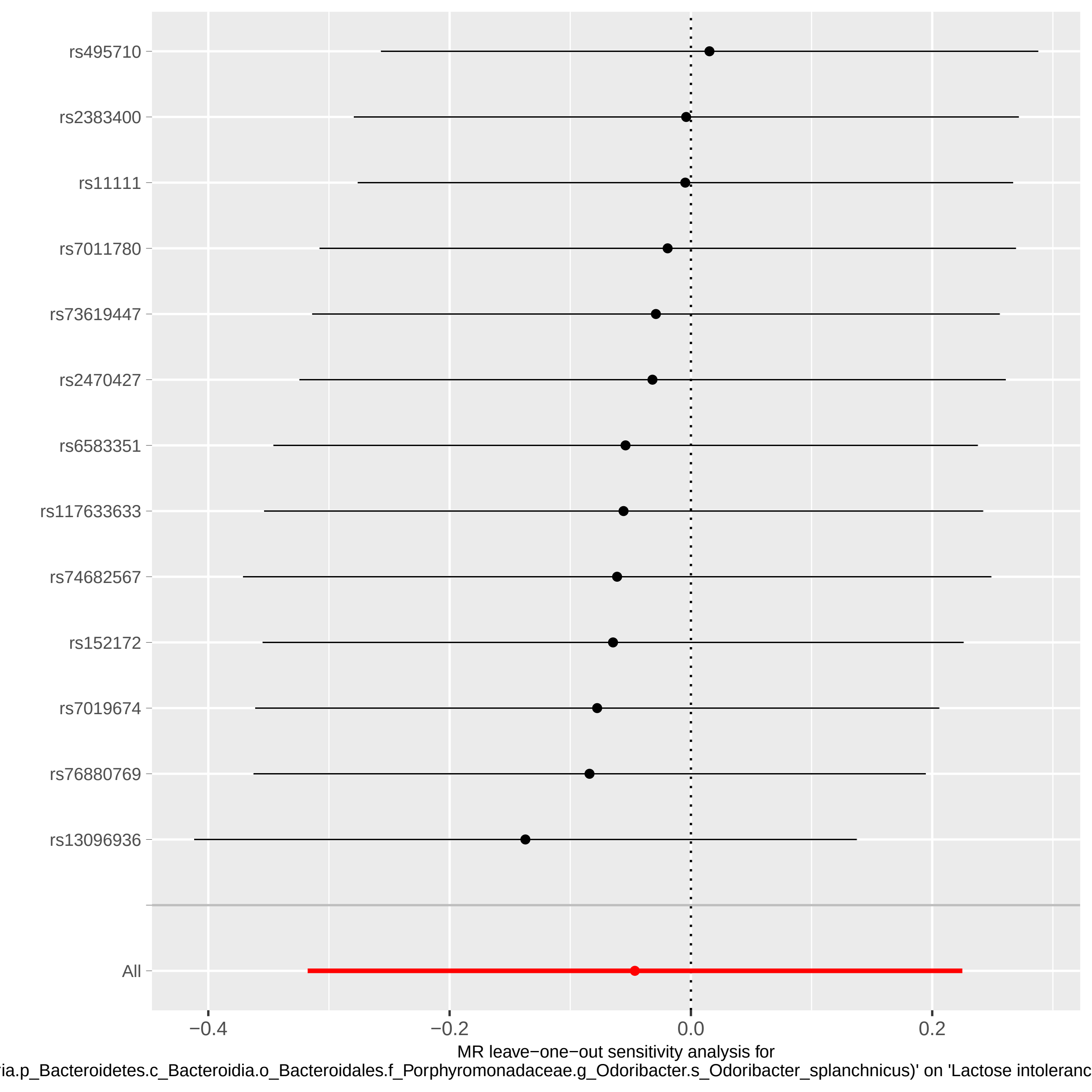

rs495710
rs2383400
rs11111
rs7011780
rs73619447
rs2470427
rs6583351
rs117633633
rs74682567
rs152172
rs7019674
rs76880769
rs13096936
All
−0.4
−0.2
0.0
0.2
MR leave−one−out sensitivity analysis for
ria.p_Bacteroidetes.c_Bacteroidia.o_Bacteroidales.f_Porphyromonadaceae.g_Odoribacter.s_Odoribacter_splanchnicus)' on 'Lactose intoleranc
